# Increased Migratory and Activation Cell Markers of Peripheral Blood Lymphocytes in an Experimental Model of Nephrotic Syndrome

**DOI:** 10.1155/2015/209764

**Published:** 2015-05-07

**Authors:** Wagner de Fátima Pereira, Gustavo Eustáquio Alvim Brito-Melo, Cláudia Martins Carneiro, Dirceu de Sousa Melo, Karine Beatriz Costa, Fábio Lourenço Tadeu Guimarães, Etel Rocha-Vieira, Érica Leandro Marciano Vieira, Ana Cristina Simões e Silva

**Affiliations:** ^1^Immunology Laboratory, Integrated Health Research Center, Universidade Federal dos Vales do Jequitinhonha e Mucuri (UFVJM), 38610-000 Diamantina, MG, Brazil; ^2^Immunopathology Laboratory, Center for Research in Biological Sciences (LIMP/NUPEB), Universidade Federal de Ouro Preto (UFOP), 30140-081 Ouro Preto, MG, Brazil; ^3^Interdisciplinary Laboratory of Medical Investigation, Pediatric Nephrology Unit, Universidade Federal de Minas Gerais (UFMG), 30130-100 Belo Horizonte, MG, Brazil

## Abstract

The present study aimed to evaluate the expression of CD80 and CD18 in subpopulations of peripheral blood leukocytes and oxidative kidney damage in rats with nephrotic syndrome (NS) induced by doxorubicin (Dox) in comparison to control animals at different time points. Male adult Wistar rats were submitted to 24-hour urine and blood collection for biochemical and immunological analysis at 7, 14, 21, and 28 days after Dox injection. After euthanasia, the kidneys were removed for histological analysis and the evaluation of oxidative stress. The phenotypic characterization of leukocytes was performed using flow cytometry. Dox-injected animals exhibited increased CD18 expression in cytotoxic T lymphocytes, NK cells, and monocytes and high CD80 expression in monocytes. Kidney oxidative damage was positively correlated with CD80 expression in monocytes and serum levels of creatinine. These results suggest that phagocytic and cytotoxic cells are preferentially recruited to the tissue injury site, which may contribute to kidney dysfunction in this animal model of NS. The blockade of integrin and costimulatory molecules may provide new therapeutic opportunities for NS.

## 1. Introduction

Nephrotic syndrome (NS) is a fairly common glomerulopathy in both children and adults characterized by proteinuria, hypoalbuminemia, generalized edema, and hyperlipidemia [1, 2]. NS can be caused by a primary renal lesion or associated with a systemic disease [1, 2]. The term idiopathic nephrotic syndrome (INS) refers to the condition caused by a primary renal lesion in which kidney histology varies from minimal podocyte changes (minimal change NS) to focal segmental glomerulosclerosis [1, 2]. Despite advances in INS studies in recent decades, the pathophysiology of this disease remains unknown.

Some studies suggest an important role of the immune system in triggering or maintaining INS, such as an abnormal T lymphocyte response and the increased release of local cytokines (see [3], for review). A tubulointerstitial inflammatory infiltrate of mononuclear cells, predominantly monocytes/macrophages and T lymphocytes, has been observed in the early stages of INS [4–6]. The intensity of the inflammatory infiltrate has been associated with a reduction in glomerular filtration, protein deposition in the extracellular matrix, scar tissue formation, and subsequent interstitial sclerosis [4, 7]. Children with focal segmental NS have more lymphocytes and macrophages in renal tissue than those with minimal change NS [6].

Kidney injury in INS may also be related to oxidative tissue damage through the increased production of reactive oxygen species (ROS) [8–10]. Recent studies have shown that patients with NS patients have higher levels of ROS than healthy controls [9]. Lipid peroxidation products have been associated with animal models of NS [8, 10, 11], while changes in the activity of superoxide dismutase (SOD) and catalase have been detected in the plasma of patients [9] and the renal tissue of animals with NS [10, 11].

As cytotoxic phagocytic cell involvement occurs in the development of kidney damage in NS [6, 7, 12] and macrophage-free radicals participate in the process [13, 14], it is likely that changes in the activation and migration of these cells influences the course of the disease. The activation of T cells requires two types of signals: (1) the connection between the T cell receptor and antigen presented by the major histocompatibility complex class II in antigen presenting cells (APCs) and (2) the costimulatory signal between the CD28 molecule on the surface of T cells and the CD80 molecule (B7-1) on the surface of APCs [15–17]. In the cell migration mechanism, leukocyte integrins and intercellular adhesion molecules play essential roles in the attachment of leukocytes to target cells and extracellular matrix [18, 19]. After injury, chemotactic signals from the inflammatory site activate integrins and induce the attachment of leukocytes and their penetration of the vascular wall [20].

Experimental models, such as chemotherapeutic induction using doxorubicin, have contributed to the understanding of the pathophysiology of NS [21]. In rodents, this drug induces kidney damage similar to that found in patients with NS [3, 22]. The aim of the present study was to investigate the expression of CD18 integrins and the costimulatory molecule CD80 in peripheral blood leukocytes associated with changes in leukocyte counts and oxidative activity in the kidneys of rats with doxorubicin-induced NS.

## 2. Methods

### 2.1. Animals

Sixty-four male Wistar rats aged six to eight weeks (body weight: 250 to 300 grams) were obtained from the animal housing facilities of the Federal University of Minas Gerais (Brazil) and kept under controlled environmental conditions with free access to food and water. The experimental protocol was approved by local animal experimentation ethics committee.

### 2.2. Induction of Nephropathy

The animals were divided into two groups: an experimental group (*n* = 32) that received a single dose (7.5 mg/Kg) of doxorubicin (Dox) (Doxolem-Farmaclinic, Belo Horizonte, Brazil) in the tail vein and a control group (*n* = 32) that received phosphate-buffered saline (PBS; 0.15 mol/L sodium chloride, 0.01 mol/L phosphate buffer at pH 7.4) under the same conditions [2, 23, 24]. Animals were euthanized under anesthesia (ketamine : xylazine, 60 : 8 mg/Kg body weight) on Days 7, 14, 21, and 28 after Dox or PBS injection. Blood and kidneys were collected for immunological and biochemical assays.

### 2.3. Kidney Function Assay

Kidney function was evaluated using 24-hour urine samples collected in metabolic cages on Days 0 (before injection), 7, 14, 21, and 28. Cobas Mira Plus was used to analyze the blood and urine samples (Roche AG, Switzerland). Commercial kits (Bioclin-Quibasa, Belo Horizonte, Brazil) were used to measure creatinine (K067) and albumin (078).

### 2.4. Peripheral Blood Leukocyte Count

Blood leukocytes were counted using a CELM CC-550 cell counter (CELM, Barueri, SP, Brazil). Differential white blood cell counts were performed in blood smears using an optical microscope (Olympus-BX41 TF, Japan) after May-Grunwald-Giemsa staining.

### 2.5. Histological and Morphometric Analyses

A portion of the kidney was used for the histological and morphometric assays. Kidney tissue was fixed in 4% paraformaldehyde (pH 7.2) for two hours and transferred to Bouin's solution for four hours. After dehydration, tissue samples were embedded in paraffin and serial horizontal sections (thickness: 4 *μ*m) were stained with hematoxylin and eosin (HE) and Gomori ammonium silver for the analysis of morphological changes and kidney fibrosis. Histological images were captured using conventional microscopy and scanned by a microcamera attached to the microscope (Leica DM5000B DFC340FX). The Image J1.43 software for Windows Vista/7 (http://rsb.info.nih.gov/ij/) was used for the analysis of the images.

### 2.6. Cell Surface Staining and Flow Cytometry Analysis

Whole peripheral blood (50 *μ*L/sample) was incubated with monoclonal antibody for the following surface markers: anti-CD3, anti-CD4, anti-CD8 (Becton & Dickinson, San Jose, CA, USA), anti-CD80, and anti-CD18 (Caltag-Medsystems Limited, Buckingham, UK) conjugated with fluorescein phycoerythrin (PE), isothiocyanate (FITC), or biotin in the dark for 30 minutes at room temperature. After incubation, the erythrocytes were lysed with Optilyse-B solution (Immunotec, USA). The cells were washed twice in 1 mL of cold PBS (pH 7.4). The biotinylated antibodies were revealed using streptavidin-FITC (Becton & Dickinson, San Jose, CA, USA) as described elsewhere [25]. Data acquisition was performed using FACScan (Becton & Dickinson, San Diego, CA, USA). Cell acquisition was processed and analyzed using the Cell Quest software (Becton & Dickinson, San Jose, CA, USA). Total T lymphocytes (CD3^+^), T helper lymphocytes (CD3^+^CD4^+^), cytotoxic T lymphocytes (CD3^+^CD8^+^), activated T lymphocyte (CD4^+^CD18^+^ and CD8^+^CD18^+^), activated monocytes (SSC^int^CD4^low+^CD18^+^ and SSC^int^CD4^low+^CD80^+^), and suggestive activated NK cells (SSC^int^CD8^low+^CD18^+^) were analyzed using fluorescence dot plots after the selection of the cell population of interest (SSC × FSC graph). These cells were then analyzed for their expression (frequency and mean fluorescent intensity (MFI)) of a given marker using histograms with markers set based on negative isotype controls.

### 2.7. Redox Status

Kidney samples were frozen in liquid nitrogen and stored at −80°C. Fragments were weighed after thawing and macerated/homogenized with PBS. Redox status was assessed using a method employed elsewhere [26]. Briefly, tissue levels of thiobarbituric acid reactive substances (TBARS), which include malondialdehyde (MDA), were determined by reaction under acidic conditions (90°C for 90 minutes) and analyzed on a microplate (532 nm). Catalase activity was determined by absorbance decreased in a hydrogen peroxide medium using a spectrophotometer (240 nm). SOD activity was calculated based on the autooxidation inhibition of pyrogallol (50%). Absorbance was measured on a microplate (420 nm). The Bradford method was used to determine the protein concentration with bovine serum albumin as the standard [26].

### 2.8. Statistical Analysis

The results were expressed as mean ± standard error of the mean, with a significance level of 95% (*p* < 0.05). Either analysis of variance (ANOVA) and Tukey's post hoc test or the Kruskal-Wallis test and the Mann-Whitney post hoc test were used for multiple comparisons within groups. Differences between groups were evaluated using either Student's *t*-test or the Mann-Whitney test, depending on the distribution of the data (normal or nonnormal). Pearson's correlation coefficients were calculated to determine the strength of associations between different variables. All analyses were performed using the Statistical Package for Social Sciences version 17.0 (SPSS, IBM, USA).

## 3. Results

### 3.1. Dox Induced Renal Changes

The biochemical results showed that the Dox group developed kidney damage, as evidenced by albuminuria and increased urine albumin/creatinine ratio (*p* < 0.05) at every time-point (Days 7 to 28) (Table 1). Previous studies have described the same response in an animal model using doxorubicin [27, 28].

### 3.2. Histological Results

**Figure 1:**
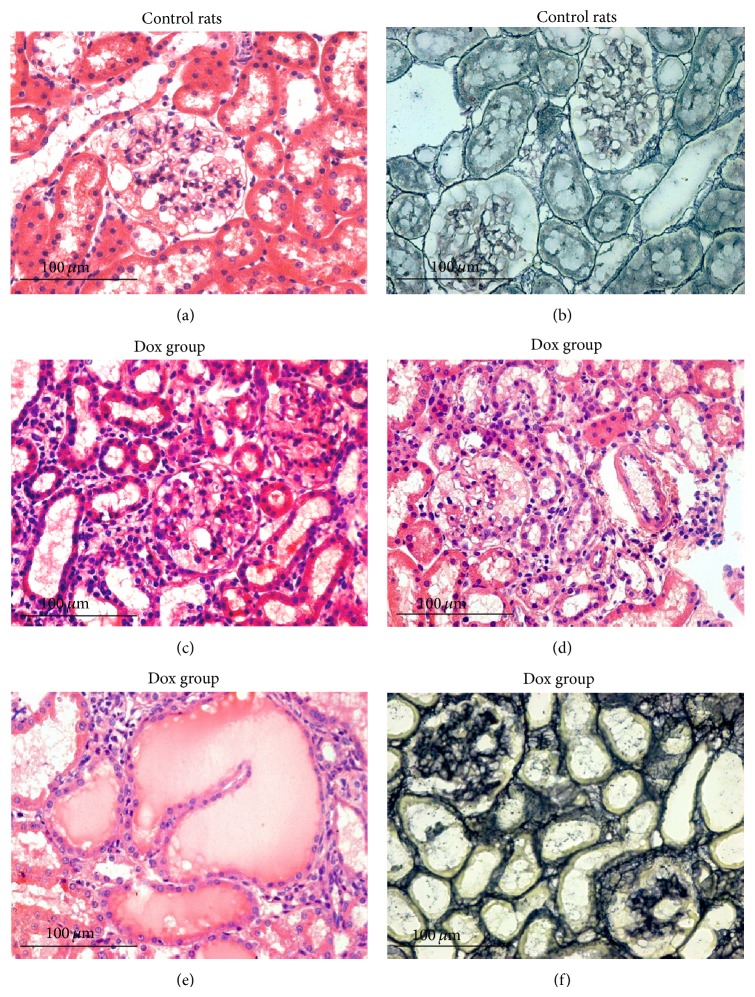
Renal histological changes in rats with doxorubicin-induced nephropathy. Control group ((a) and (b)): normal tissue appearance (HE and Gomori ammonium silver, resp.); Dox group ((c) and (d)) Day 28 after doxorubicin injection: reduction in glomerular cellularity and increased tubulointerstitial cellularity (HE). Dox Group ((e) and (f)) on Day 28: areas of hyaline deposits and interstitial expansion (HE and ammonia Silver, resp.). Focal glomerulosclerosis with partial obliteration of glomerular capillaries in (f) (Gomori ammonium silver). Scale bar 100 *μ*m.

At Day 28 after the injection of Dox, a reduction in glomerular cells and an increased tubular interstitial cellularity were found (Figure 1). Gomori ammonium silver staining revealed interstitial expansion in the Dox group (Figure 1(f)), demonstrated by the increased thickness of the basement membrane of the tubules and glomeruli and signs of focal segmental glomerulosclerosis and characterized by partial obliteration of glomerular capillaries (Figures 1(e) and 1(f)).

### 3.3. Frequency of Leukocyte Subpopulation in Peripheral Blood

In the early stage of the disease (Day 7 after injection), a significant reduction in the total leukocyte count was found in the Dox group in comparison to the control group (Table 1), especially regarding the percentage of monocytes and neutrophils (Table 1). Interestingly, in the intermediate phase (Day 14 after injection), the leukocyte production was recovered and increased in the Dox group in comparison to the control group (Table 1), with an increase in the number of monocytes and neutrophils, but a reduction in the number of lymphocytes (Table 1). The total amount of leukocytes remained increased in the late stages of the disease (Days 21 to 28 after injection) in the Dox group, but with no significant difference when compared to the control group (Table 1). The percentage of peripheral blood monocytes and lymphocytes in the Dox group increased at Day 21 and decreased at Day 28, while the percentage of neutrophils remained increased from Days 14 to 28 (Table 1). To gain better understanding of the immune cell response in NS, flow cytometry was employed and demonstrated an increase in the frequency of cytotoxic T lymphocytes (CD3^+^CD8^+^) and helper T lymphocytes (CD3^+^CD4^+^) in the early stage (Day 7 after injection) in the Dox group (Table 1).

### 3.4. Prevalence of Activated T CD8^+^ Lymphocytes, NK, and Monocytes in Blood of Animals in Dox Group

**Figure 2:**
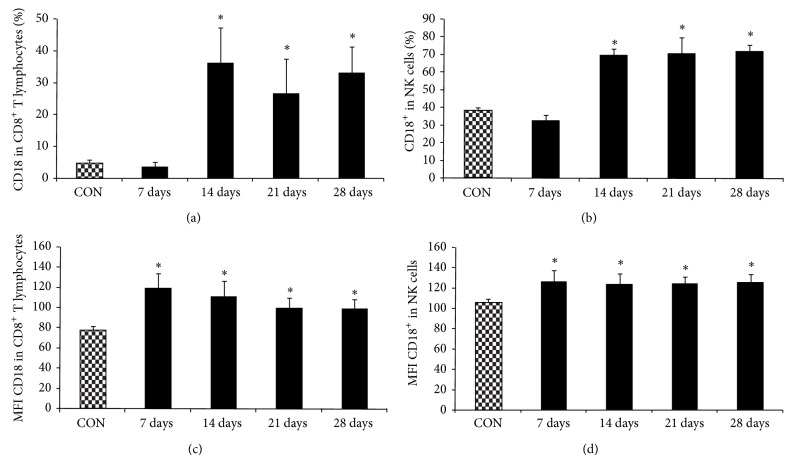
CD18 expression in CD8^+^ T lymphocytes and NK cells from rats with nephrotic syndrome induced by doxorubicin and control animals in* ex vivo* condition. Blood leukocytesin rats (*n* = 32) that received a single dose of doxorubicin in the tail vein (7.5 mg/kg) and control rats (*n* = 32) that received PBS in the same condition were stained* ex vivo *for CD18 and CD8 expression. Data were collected using flow cytometry and analyzed using CellQuest software. Graphs (a) % of CD18^+^CD8^+^, (b) % of CD8^low+^CD18^+^, (c) MFI of CD18^+^CD8^+^, and (d) MFI of CD8^low+^CD18^+^ show expression of the given marker in lymphocytes. The data expressed as mean ±  standard error for Dox group (black bars) and control group (grew bars). ^∗^ *p* < 0.05 for the comparison between Dox and control group at the same time-point (Student's *t*-test).

**Figure 3:**
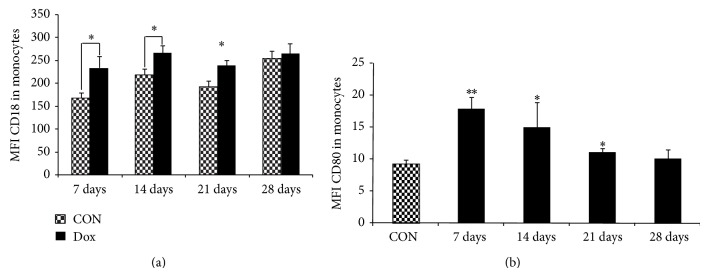
CD18 and CD80 expression in total monocytes from rats with nephrotic syndrome induced by doxorubicin and control animals in* ex vivo* condition. Blood leukocytesfrom rats (*n* = 32) that received a single dose of doxorubicin in tail vein (7.5 mg/kg) and control rats (*n* = 32) that received PBS in the same condition were stained* ex vivo *for CD18 and CD80 expression. Data were collected using flow cytometry and analyzed using CellQuest software. Graphs (a) MFI of CD18^+^ monocytes and (b) MFI of CD80^+^ monocytes show expression of the given marker in lymphocytes. The data expressed as mean ±  standard error for Dox group (black bars) and control group (grew bars). ^∗^ *p* < 0.05 for the comparison between Dox and control group at the same time-point (Student's *t*-test).

Costimulatory molecules, such as CD18 and CD80, work together to induce regulated adaptive T cell and B cell responses by binding to receptors on their surface and recruiting neutrophils and other leukocytes. Regarding the state of cell activation by CD18 expression, in all phases (Days 7 to 28), the frequency of CD8^+^CD18^+^ T cells and mean fluorescence intensity (MFI) were increased in the Dox group in comparison to the control group (Figures 2(a) and 2(b)). The frequency of NK cells (SSC^int^CD8^low+^CD18^+^ cells) and MFI were also increased in the Dox group (Figures 2(c) and 2(d)) in comparison to the control group. Monocytes in the Dox group also had increased CD18 and CD80 expression (Figures 3(a) and 3(b)). In monocytes, a slight reduction in the expression of CD18 and CD80 was found at Day 21 in comparison to the early stages (Days 7 and 14), but remained significantly higher in comparison to the control group (Figures 3(a) and 3(b)). On Day 28, no difference was found in CD18 and CD80 expression in monocytes (Figures 3(a) and 3(b)). There was no significant difference in CD18 expression in neutrophils between groups (data not shown).

### 3.5. Correlations among Lipid Peroxidation, Antioxidant Activity in Kidney Tissue, and Costimulatory Molecules in Dox Group

Lipid peroxidation is a well-defined mechanism of cell damage that occurs* in vivo* in different disease states. Lipid peroxides are unstable markers of oxidative stress that decompose to form complex, reactive by-products. In the initial stage (Day 7), increased lipid peroxidation (MDA) was found in the kidney tissue in the Dox group in comparison to the control group (Table 1). However, a progressive reduction in MDA concentration in kidney tissue occurred in the Dox group from Days 14 to 28 (Table 1). The antioxidant activity of SOD and catalase in kidney tissue was also evaluated and did not differ significantly between groups (Table 1). Correlation analyses were performed to gain insights into the redox state, antioxidant activity, and costimulatory molecule. Interestingly, positive correlations were found between MDA and renal parameters, such as catalase activity in kidney tissue, serum creatinine concentration, and CD80 expression in peripheral blood monocytes (Table 2).

## 4. Discussion

Experimental models contribute to the understanding of pathophysiological mechanisms and therapeutic approaches for NS [21, 29]. One such model involves the induction of nephropathy using doxorubicin [3, 22, 29]. In the present study, animals injected with doxorubicin exhibited albuminuria and an increased albumin/creatinine ratio, which is in agreement with data described in the literature [27, 30]. Albuminuria is a biochemical marker of primary renal damage [31] and a good indicator of NS in animal models [27]. The albumin/creatinine ratio in urine is also used to assess kidney function [28, 32]. In the present study, the animals in the Dox group exhibited alterations in the expression of molecules related to the cell activation (CD80) and migration (CD18) in peripheral blood leukocytes as well as an increase in MDA concentration in kidney tissue, suggesting greater oxidative damage tissue, with a positive correlation found between CD80 expression in monocytes and creatinine plasma levels.

The costimulatory molecule CD80 is a transmembrane protein found on the surface of APCs, which have affinity for CD28 molecules on the surface of T lymphocytes [15–17]. Integrins are ligand-specific transmembrane glycoproteins distributed on the cell surface that play a key role in leukocyte attachment and have an affinity for extracellular matrix, soluble ligands, and counterreceptors on endothelial cells [33, 34]. These integrins form heterodimers with covalent bonds between the alpha and beta subunits, constituting four families [35]. The beta-2 integrin family is comprised of beta subunit 2 (*β*2) and denominated CD18, in combination with one of the four alpha subunits: *α*L (CD11a), *α*M (CD11b), *α*X (CD11c), or *α*D (CD11d) [19]. Following injury, chemotactic signals activate the expression of integrins and induce the attachment of leukocytes and their penetration of the vascular wall [20]. Beta-2 integrin binds to intercellular adhesion molecule-1 (ICAM-1) on the surface of endothelial cells [36]. Cytokines and chemokines increase the expression of ICAM-1 on the vascular endothelium surface and activate integrins, inducing cell attachment and diapedesis on the surface of leukocytes [37]. Thus, increased integrin expression is directly related to the cell activation mechanisms and migration [18].

A reduction in peripheral blood leukocytes was found in the Dox group in the early phase of NS when compared to the control group, with decreased expression of monocyte subpopulations and neutrophils. Such changes may be related to the myelosuppressive effect of acute drug-induced NS, which induces reversible leukopenia and neutropenia [38]. Another possibility would be the early increase in the migratory capacity of activated cells, with the high expression of the costimulatory molecule CD80 and the integrin CD18, especially on the 7th day after doxorubicin injection. We are not able to prove with certainty that the effects of doxorubicin were only due to renal injury and not due to an indirect hematological effect. However, it is very unlikely that the effects of doxorubicin observed in our study were exclusively due to hematological toxicity. We should take into account that, in rats and mice, doxorubicin is very rapidly removed from the plasma after injection and deposited in tissues, especially in the kidney [38]. It is well known that a single and low dose of doxorubicin is sufficient to produce kidney tissue accumulation and histological pattern of focal segmental glomerulosclerosis (see, for review, [39, 40]). Furthermore, the early presence of macrophages in renal tissue and the association of this finding with renal damage have been well characterized in doxorubicin-induced nephropathy [41–43].

MDA concentration in the kidney tissue increased in the Dox group in the initial stage, suggesting greater oxidative tissue damage. Previous studies report the involvement of cytotoxic and phagocytic cells in the development of kidney damage in NS [6, 7, 12], as the activation of macrophages and neutrophils can result in the generation of free radicals [13, 14, 44]. The change in leukocyte activation and migration may influence the course of NS, with the possible participation of these cells in renal oxidative stress and albuminuria induction in the early stage of the disease. Human patients with NS exhibit increased ROS production in the plasma associated with a reduction in the amount of plasma albumin [9]. In an animal model of doxorubicin-induced NS, increased TBARS production has been found [8], associated with a reduction in the activity of catalase in renal tissue [11]. Albuminuria and lipid peroxidation have also been associated with kidney damage in doxorubicin-induced NS [10]. The results of the present study reveal peaks in urinary albumin excretion and MDA production seven days after doxorubicin injection, suggesting the possible involvement of ROS in the initial change in the glomerular filtration membrane.

Oxidative damage in the kidney has also been associated with the lower activity of the antioxidant enzymes superoxide dismutase (SOD) and catalase [9–11]. In the plasma of patients with NS, higher ROS production is related to a decrease in SOD activity [9]. Boonsanit and colleagues demonstrated an increase in plasma levels of TBARS and decrease in catalase levels in renal tissue in rats with doxorubicin-induced NS [11]. Glomerulosclerosis, albuminuria, and lipid peroxidation in renal tissue were found to be more severe in mice deficient in catalase than in wild-type mice after doxorubicin injection [10]. However, in the present study, the increase in renal levels of MDA did not induce significant changes in SOD or catalase activity, although there was a mild elevation in the activity of renal catalase at Day 7. This increased enzyme activity might probably be responsible for the correlation, even though weak, between elevated lipid peroxidation and an antioxidant renal response. This finding may also indicate that the increased MDA production was probably not due to reduced antioxidant activity.

Although the present findings indicate the possible involvement of peripheral monocytes/macrophages in renal oxidative damage and since the literature indicates the involvement of ROS in the pathogenesis of NS [9, 13], this situation should be evaluated with caution. It is important to consider the action mechanism of doxorubicin, which can also induce the formation of ROS in the kidneys [45], favoring initial tissue damage. However, one should not underestimate the importance of the immune system, especially the role of monocytes/macrophages, in kidney damage. Research has shown that the macrophage phenotype (either M1 or M2) is directly related to the worsening or improvement, respectively, of kidney damage in doxorubicin-induced NS [4, 5]. Moreover, greater macrophage infiltration in renal tissue in children with NS is related to a worse prognosis [6].

The expression of CD80 and CD18 in monocytes was higher in the first two weeks and not statistically significant in the fourth week after injection. This monocyte activation has also been demonstrated in previous studies, with the early accumulation of macrophages in the renal tissue using the same animal model for NS [29, 46, 47]. Moreover, a positive correlation has been found between the expression of CD18/ICAM-1 in kidney tissue and local macrophages infiltrate in NS induced by puromycin aminonucleoside, with cellular infiltration kinetics and integrin expression similar in the 2nd week, returning to normal in the 7th week after injection [48].

**Figure 4:**
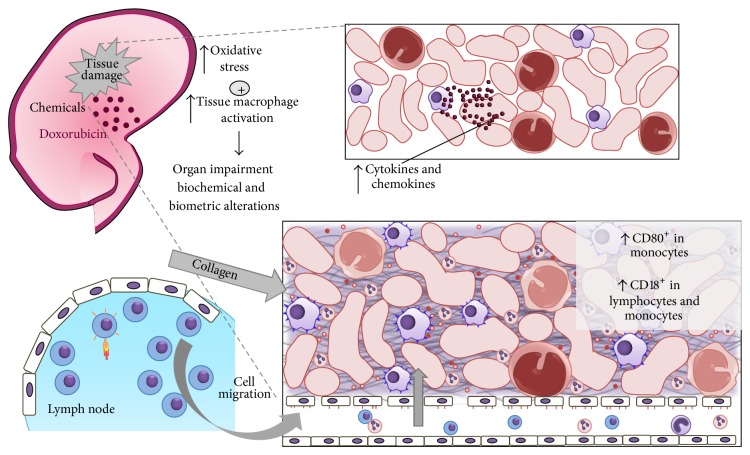
Schematic figure of proposed pathogenesis of kidney damage in doxorubicin-induced nephropathy. Doxorubicin induced chemical kidney damage, which activated tissue macrophages and increased local oxidative stress. This situation caused organ impairment and biochemical alterations. Activated macrophages released cytokines and chemokines, which activated and recruited new cells, especially monocytes and lymphocytes from lymph nodes. These cells accumulated in the kidneys, inducing collagen accumulation and, consequently, worsened kidney damage.

The increase in the frequency of peripheral lymphocytes in the early stage of NS occurred for cytotoxic T lymphocytes (CD3^+^CD8^+^) and T helper cells (CD3^+^CD4^+^). However, CD18 expression was higher in cytotoxic T lymphocytes and NK cells throughout the experimental period, suggesting greater migratory activity, especially after the 14th day. These findings are in agreement with previous studies that report changes in the activity of NK cells [7, 12] and cytotoxic T lymphocytes in patients with NS [12, 49, 50] as well as animals models of doxorubicin-induced NS [2, 3, 51]. Furthermore, proteinuria has been associated with mononuclear cell infiltrate and the expression of CD18 and ICAM-1 in kidney tissue [52]. The greater migratory activity of lymphocytes was also confirmed by the reduction in the frequency of these cells in the peripheral blood 14 days after doxorubicin injection. A previous study detected lymphocytes in the kidney tissue of animals with doxorubicin-induced NS in a later stage following the arrival of macrophages [3]. The increased migration of cytotoxic T lymphocytes and NK cells may also be related to collagen deposition in kidney tissue in the 3rd and 4th week after doxorubicin injection (Figures 1 and 4).

The increased frequency of lymphocytes in the early phase of NS may have only been a reflection of the reduction in the frequency of monocytes and neutrophils and not an absolute increase in lymphocytes. Likewise, the reduction in lymphocytes on Day 14 may also have only been a reflection of the increase in monocytes and neutrophils. However, the Dox group demonstrated leukocytosis on Day 14, reflecting a possible immune response exacerbation due to kidney damage. In an animal model of glomerulonephritis, it was evident that CD80 plays a detrimental role in kidney disease by promoting CD4^+^ survival and proliferation [53]. According to Wilson and colleagues, the CD80 molecule is linked to the activation of NK cells [54], which could explain the increased expression of CD18 in NK cells in the present study.

The importance of neutrophils to kidney damage in an animal model of doxorubicin-induced NS has been underinvestigated. However, studies have shown greater neutrophil oxidative activity in patients with NS [13, 44]. In the present study, despite a reduction in the neutrophil subpopulation in the initial stage, there was no significant change in CD18 expression. However, neutrophil migration is dependent not only on CD18 [55], but also on other molecules, such as P-selectin glycoprotein ligand 1 [56–58] and L-selectin [58].

We are aware of the limitations of our study. Indeed, our findings did not allow us to show causality relation between renal damage and changes in the profile of peripheral lymphocytes associated with local oxidative stress. Alternatively, we have only pointed out to a potential role of increased MDA levels, higher CD18 expression in cytotoxic T lymphocytes, NK cells and monocytes, and higher CD80 expression in monocytes in renal damage induced by doxorubicin. In addition, we did not evaluate temporal changes in macrophage/monocyte numbers, especially in classical macrophages (M-1) and alternative macrophages (M-2) subpopulations, in renal tissue. It is well recognized that macrophages can contribute extensively to renal tissue damage through a number of mechanisms, including their production of proinflammatory cytokines and their T cell stimulatory capacity [42, 43, 59]. Tissue factors determine the phenotype of monocytes/macrophages recruited into the renal tissue, whereas the profile of locally released cytokines regulates the differentiation of mononuclear cells. Th1-type cytokines induce differentiation into classical macrophages, denominated M-1, that produce cytotoxic and proinflammatory cytokines, while Th2-type cytokines induce alternative macrophages, denominated M-2, responsible for the synthesis of anti-inflammatory cytokines [43, 59]. The characterization of the phenotype of macrophages would be important to support the correlation between renal function impairment and MDA levels.

Nevertheless, our findings at least suggest a cell migration profile involving phagocytosis and cytotoxicity at different times in the progression of doxorubicin-induced nephropathy. The intense activation of monocytes in the early stages of NS suggest, at least in part, a potential role for these cells in pathogenesis of kidney damage, which could represent a relationship with oxidative kidney damage, as hypothesized in Figure 4. Thus, due to the importance of the costimulatory process during the activation of immune cells [53], characterizing the temporal expression of different molecules related to cell activation and migration may contribute to future immune therapies in renal tissue, thereby preventing the perpetuation of the tissue damage in patients with NS. Further studies are obviously necessary to investigate temporal cellular changes in renal tissue, the precise role of M-1 and M-2 macrophages, and the sequence of local tissue events elicited by doxorubicin-induced NS.

## 5. Conclusion

Doxorubicin-induced NS is characterized by the increased expression of CD80 in monocytes and CD18 in monocytes, CD8 lymphocytes, and NK cells, especially in the early stages of the disease. The increased expression of these molecules related to the activation and migration of immune cells may contribute to the pathogenesis of kidney damage. Moreover, oxidative damage in the kidney was positively correlated with CD80 expression in monocytes and serum creatinine. These findings indicate an association between monocyte activation and kidney damage in NS. Further studies evaluating the blockade of integrins and costimulatory molecules as well as temporal changes on circulating cells populations and possible alterations on the phenotype of macrophages in renal tissue may offer new therapeutic opportunities for patients with NS.

## Figures and Tables

**Table 1:** Urinary protein excretion, blood cells, and markers of cell surface and of *redox status* in the kidney of rats with doxorubicin-induced nephropathy and controls.

	Control Group	Doxorubicin goup			
		T-07	T-14	T-21	T-28
	Mean (SE)	Mean (SE)	Mean (SE)	Mean (SE)	Mean (SE)
Albuminuria (mg/L)	62.92 (18.09)	122.66 (23.17)^∗^	102.43 (16.57)^∗^	87.05 (3.43)^∗^	79.06 (7.90)^∗^
Urine albumin/creatinine ratio (mg/g)	0.39 (0.07)	0.92 (0.18)^∗^	1.05 (0.35)^∗^	1.09 (0.41)^∗^	0.98 (0.11)^∗^
WBC × 1000	6.81 (0.58)	3.14 (0.14)^∗^	8.37 (0.82)^∗^	10.87 (1.09)	7.5 (0.96)
Monocytes (%)	6.07 (0.52)	4.19 (0.70)^∗^	7.19 (2.40)	7.13 (0.76)^∗^	3.20 (0.78)^∗^
Neutrophil (%)	14.45 (1.16)	10.00 (1.29)^∗^	34.77 (3.16)^∗^	20.87 (3.94)^∗^	33.70 (4.25)^∗^
Lymphocyte (%)	79.17 (2.45)	85.49 (1.41)^∗^	57.24 (4.59)^∗^	71.55 (2.23)^∗^	62.50 (4.40)^∗^
Eosinophil (%)	0.18 (0.06)	0.20 (0.13)	0.10 (0.10)	0.26 (0.21)	0.30 (0.21)
Basophil (%)	0.13 (0.11)	0.11 (0.11)	0.70 (0.30)	0.18 (0.13)	0.30 (0.21)
Monocytes × 1000	0.414 (0.185)	0.132 (0.053)^∗^	0.602 (0.783)	0.775 (0.257)^∗^	0.240 (0.174)^∗^
Neutrophil × 1000	0.984 (0.465)	0.314 (0.084)^∗^	2.910 (1.375)^∗^	2.269 (1.074)^∗^	2.528 (1.414)^∗^
Lymphocyte × 1000	5.391 (1.415)	2.684 (0.304)^∗^	4.791 (1.053)^∗^	7.778 (1.294)^∗^	4.688 (0.618)^∗^
Eosinophil × 1000	0.012 (0.024)	0.000 (0.00)	0.008 (0.019)	0.029 (0.050)	0.023 (0.048)
Basophil × 1000	0.009 (0.018)	0.003 (0.01)	0.059 (0.093)	0.019 (0.046)	0.023 (0.056)
T cell CD3^+^CD4^+^ (%)	45.94 (1.70)	79.92 (1.92)^∗^	57.53 (2.03)	47.93 (4.30)	47.86 (3.52)
T cell CD3^+^CD8^+^ (%)	13.41 (0.55)	16.19 (1.00)^∗^	12.09 (1.74)	12.70 (0.92)	13.40 (0.88)
T cell CD4^+^CD18^+^ (MFI)	10.72 (0.52)	11.54 (1.18)	12.43 (0.57)	12.65 (1.60)	12.02 (1.07)
MDA (mmol/mg protein)	2.18 (0.11)	3.32 (0.51)^∗^	2.62 (0.40)	2.09 (0.52)	1.80 (0.22)
Catalase (ΔE/min/mg protein).	4.61 (0.37)	5.83 (1.63)	4.97 (0.89)	4.94 (0.91)	4.21 (0.47)
SOD (U/mg protein)	0.39 (0.02)	0.36 (0.04)	0.32 (0.02)	0.42 (0.02)	0.38 (0.02)

MDA: malondialdehyde; SOD: superoxide dismutase.

^∗^ *p* < 0.05 compared to control group.

**Table 2:** Correlations of redox status in kidney tissue, renal function parameters, and immune markers in rats with nephrotic syndrome induced by doxorubicin.

Dox group	MDA	
	*r*	*p*
SOD	0.213	0.241
Catalase	0.382	0.031^∗^
Plasma creatinine levels	0.535	0.009^∗^
Urinary creatinine levels	0.170	0.225
Albuminuria	0.124	0.277
Monocytes CD80 (MFI)	0.411	0.015^∗^
Monocytes CD18 (MFI)	0.254	0.110

MDA: malondialdehyde; SOD: superoxide dismutase; MFI: mean fluorescence intensity.

^∗^ *p* < 0.05 compared to control group.

## References

[1] D'Agati V. D., Kaskel F. J., Falk R. J. (2011). Focal segmental glomerulosclerosis. *The New England Journal of Medicine*.

[2] Wang Y., Wang Y., Feng X. (2001). Depletion of CD4^+^ T cells aggravates glomerular and interstitial injury in murine adriamycin nephropathy. *Kidney International*.

[3] Wang Y., Wang Y. P., Tay Y.-C., Harris D. C. H. (2000). Progressive adriamycin nephropathy in mice: sequence of histologic and immunohistochemical events. *Kidney International*.

[4] Cao Q., Wang Y., Zheng D. (2010). IL-10/TGF-beta-modified macrophages induce regulatory T cells and protect against adriamycinnephrosis. *Journal of the American Society of Nephrology*.

[5] Wang Y., Wang Y. P., Zheng G. (2007). *Ex vivo* programmed macrophages ameliorate experimental chronic inflammatory renal disease. *Kidney International*.

[6] Benz K., Büttner M., Dittrich K., Campean V., Dötsch J., Amann K. (2010). Characterisation of renal immune cell infiltrates in children with nephrotic syndrome. *Pediatric Nephrology*.

[7] Musiał K., Ciszak L., Kosmaczewska A., Szteblich A., Frydecka I., Zwolińska D. (2010). Zeta chain expression in T and NK cells in peripheral blood of children with nephrotic syndrome. *Pediatric Nephrology*.

[8] Zima T., Tesar V., Crkovska J. (1998). ICRF-187 (dexrazoxan) protects from adriamycin-induced nephrotic syndrome in rats. *Nephrology Dialysis Transplantation*.

[9] Ghodake S. R., Suryakar A. N., Ankush R. D., Shaikh K., Katta A. V. (2010). Role of reactive oxygen species in pathogenesis of nephrotic syndrome. *Indian Journal of Clinical Biochemistry*.

[10] Takiue K., Sugiyama H., Inoue T. (2012). Acatalasemic mice are mildly susceptible to adriamycin nephropathy and exhibit increased albuminuria and glomerulosclerosis. *BMC Nephrology*.

[11] Boonsanit D., Kanchanapangka S., Buranakarl C. (2006). L-carnitine ameliorates doxorubicin-induced nephrotic syndrome in rats. *Nephrology (Carlton)*.

[12] Lama G., Luongo I., Tirino G., Borriello A., Carangio C., Salsano M. E. (2002). T-lymphocyte populations and cytokines in childhood nephrotic syndrome. *American Journal of Kidney Diseases*.

[13] Akyol T., Bulucu F., Sener O. (2007). Functions and oxidative stress status of leukocytes in patients with nephrotic syndrome. *Biological Trace Element Research*.

[14] Berens K. L., Verani R. R., Luke D. R. (1998). Role of neutrophils and macrophages in experimental nephrosis of the rat. *Renal Failure*.

[15] Engel P., Gribben J. G., Freeman G. J. (1994). The B7-2 (B70) costimulatory molecule expressed by monocytes and activated B lymphocytes is the CD86 differentiation antigen. *Blood*.

[16] Fleischer J., Soeth E., Reiling N., Grage-Griebenow E., Flad H.-D., Ernst M. (1996). Differential expression and function of CD80 (B7-1) and CD86 (B7-2) on human peripheral blood monocytes. *Immunology*.

[17] Sayegh M. H., Turka L. A. (1995). T cell costimulatory pathways: promising novel targets for immunosuppression and tolerance induction. *Journal of the American Society of Nephrology*.

[18] Gahmberg C. G. (1997). Leukocyte adhesion: CD11/CD18 integrins and intercellular adhesion molecules. *Current Opinion in Cell Biology*.

[19] Springer T. A. (1994). Traffic signals for lymphocyte recirculation and leukocyte emigration: the multistep paradigm. *Cell*.

[20] Penberthy T. W., Jiang Y., Graves D. T. (1997). Leukocyte adhesion molecules. *Critical Reviews in Oral Biology and Medicine*.

[21] Eddy A. A., López-Guisa J. M., Okamura D. M., Yamaguchi I. (2012). Investigating mechanisms of chronic kidney disease in mouse models. *Pediatric Nephrology*.

[22] Lee V. W., Harris D. C. (2011). Adriamycin nephropathy: a model of focal segmental glomerulosclerosis. *Nephrology*.

[23] Park E.-S., Kim S.-D., Lee M.-H. (2003). Protective effects of N-acetylcysteine and selenium against doxorubicin toxicity in rats. *Journal of Veterinary Science*.

[24] Rangan G. K., Wang Y., Tay Y.-C., Harris D. C. H. (1999). Inhibition of nuclear factor-kappaB activation reduces cortical tubulointerstitial injury in proteinuric rats. *Kidney International*.

[25] Wu H., Wang Y. M., Hu M. (2007). Depletion of *γδ* T cells exacerbates murine adriamycin nephropathy. *Journal of the American Society of Nephrology*.

[26] Barreto T. O., Cleto L. S., Gioda C. R. (2012). Swim training does not protect mice from skeletal muscle oxidative damage following a maximum exercise test. *European Journal of Applied Physiology*.

[27] Pippin J., Kumar V., Stein A., Jablonski P., Shankland S. J., Davis C. L. (2009). The contribution of podocytes to chronic allograft nephropathy. *Nephron—Experimental Nephrology*.

[28] Jeansson M., Björck K., Tenstad O., Haraldsson B. (2009). Adriamycin alters glomerular endothelium to induce proteinuria. *Journal of the American Society of Nephrology*.

[29] Lee V. W. S., Wang Y., Qin X. (2006). Adriamycin nephropathy in severe combined immunodeficient (SCID) mice. *Nephrology Dialysis Transplantation*.

[30] Bertani T., Poggi A., Pozzoni R. (1982). Adriamycin-induced nephrotic syndrome in rats: sequence of pathologic events. *Laboratory Investigation*.

[31] Thomas M. E., Brunskill N. J., Harris K. P. G. (1999). Proteinuria induces tubular cell turnover: a potential mechanism for tubular atrophy. *Kidney International*.

[32] Chen A., Wei C.-H., Sheu L.-F., Ding S.-L., Lee W.-H. (1995). Induction of proteinuria by adriamycin or bovine serum albumin in the mouse. *Nephron*.

[33] Diamond M. S., Springer T. A. (1994). The dynamic regulation of integrin adhesiveness. *Current Biology*.

[34] Ruoslahti E. (1991). Integrins. *The Journal of Clinical Investigation*.

[35] Humphries J. D., Byron A., Humphries M. J. (2006). Integrin ligands at a glance. *Journal of Cell Science*.

[36] Li R., Xie J., Kantor C. (1995). A peptide derived from the intercellular adhesion molecule-2 regulates the avidity of the leukocyte integrins CD11b/CD18 and CD11c/CD18. *Journal of Cell Biology*.

[37] Carr M. W., Alon R., Springer T. A. (1996). The C-C chemokine MCP-1 differentially modulates the avidity of beta 1 and beta 2 integrins on T lymphocytes. *Immunity*.

[38] Yesair D. W., Schwartzbach E., Shuck D., Denine E. P., Asbell M. A. (1972). Comparative pharmacokinetics of daunomycin and adriamycin in several animal species. *Cancer Research*.

[39] Lee V. W. S., Harris D. C. H. (2011). Adriamycin nephropathy: a model of focal segmental glomerulosclerosis. *Nephrology*.

[40] Pereira W. F., Brito-Melo G. E., de Almeida C. A. (2015). The experimental model of nephrotic syndrome induced by Doxorubicin in rodents: an update. *Inflammation Research*.

[41] Kuncio G. S., Neilson E. G., Haverty T. (1991). Mechanisms of tubulointerstitial fibrosis. *Kidney International*.

[42] Le Berre L., Hervé C., Buzelen F., Usal C., Soulillou J.-P., Dantal J. (2005). Renal macrophage activation and Th2 polarization precedes the development of nephrotic syndrome in Buffalo/Mna rats. *Kidney International*.

[43] Cao Q., Wang Y., Zheng D. (2010). IL-10/TGF-*β*-modified macrophages induce regulatory T cells and protect against adriamycin nephrosis. *Journal of the American Society of Nephrology*.

[44] Bertelli R., Trivelli A., Magnasco A. (2010). Failure of regulation results in an amplified oxidation burst by neutrophils in children with primary nephrotic syndrome. *Clinical & Experimental Immunology*.

[45] Barbey M.-M., Fels L. M., Soose M. (1989). Adriamycin affects glomerular renal function: evidence for the involvement of oxygen radicals. *Free Radical Research Communications*.

[46] Vielhauer V., Berning E., Eis V. (2004). CCR1 blockade reduces interstitial inflammation and fibrosis in mice with glomerulosclerosis and nephrotic syndrome. *Kidney International*.

[47] Wu H., Wang Y., Tay Y.-C. (2005). DNA vaccination with naked DNA encoding MCP-1 and RANTES protects against renal injury in adriamycin nephropathy. *Kidney International*.

[48] Fernandez L., Romero M., Rincón J., Mosquera J. (2003). Increased expression of CD54, CD18, MHC class II molecules, and proliferating cell nuclear antigen in acute puromycin aminonucleoside nephrosis. *Nephron Experimental Nephrology*.

[49] Fiser R. T., Arnold W. C., Charlton R. K., Steele R. W., Childress S. H., Shirkey B. (1991). T-lymphocyte subsets in nephrotic syndrome. *Kidney International*.

[50] Herrod H., Stapleton F. B., Trouy R. L., Roy S. (1983). Evaluation of T lymphocyte subpopulations in children with nephrotic syndrome. *Clinical & Experimental Immunology*.

[51] Wang Y., Wang Y. P., Tay Y. C., Harris D. C. H. (2001). Role of CD8^+^ cells in the progression of murine adriamycin nephropathy. *Kidney International*.

[52] Wu J.-C., Fan G.-M., Kitazawa K., Sugisaki T. (1996). The relationship of adhesion molecules and leukocyte infiltration in chronic tubulointerstitial nephritis induced by puromycin aminonucleoside in Wistar rats. *Clinical Immunology and Immunopathology*.

[53] Odobasic D., Kitching A. R., Tipping P. G., Holdsworth S. R. (2005). CD80 and CD86 costimulatory molecules regulate crescentic glomerulonephritis by different mechanisms. *Kidney International*.

[54] Wilson J. L., Charo J., Martín-Fontecha A. (1999). NK cell triggering by the human costimulatory molecules CD80 and CD86. *The Journal of Immunology*.

[55] Jannat R. A., Robbins G. P., Ricart B. G., Dembo M., Hammer D. A. (2010). Neutrophil adhesion and chemotaxis depend on substrate mechanics. *Journal of Physics Condensed Matter*.

[56] Moore K. L., Patel K. D., Bruehl R. E. (1995). P-selectin glycoprotein ligand-1 mediates rolling of human neutrophils on P-selectin. *The Journal of Cell Biology*.

[57] Panés J., Perry M., Granger D. N. (1999). Leukocyte-endothelial cell adhesion: avenues for therapeutic intervention. *British Journal of Pharmacology*.

[58] De Rossi L. W., Horn N. A., Buhre W., Gass F., Hutschenreuter G., Rossaint R. (2002). The effect of isoflurane on neutrophil selectin and *β*2-integrin activation in vitro. *Anesthesia and Analgesia*.

[59] Pereira W. D. F., Brito-Melo G. E. A., Guimarães F. T. L., Carvalho T. G. R., Mateo E. C., E Silva A. C. S. (2014). The role of the immune system in idiopathic nephrotic syndrome: a review of clinical and experimental studies. *Inflammation Research*.

